# Letter from Adjunct Professor Martins, *of the Faculty of Medicine of Paris*

**Published:** 1839-06-01

**Authors:** 


### FOREIGN CORRESPONDENCE.

### LETTER FROM ADJUNCT PROFESSOR MARTINS, *of the Faculty of Medicine of Paris.*

No. I.

*Professor Trousseau's Views on the Italian Doctrine of Counter-Stimulation.*

To the Editors of the Medical Examiner.

Paris, 23*d April*, 1839.

By the death of the late Professor Alibert, the chair of Materia Medica and Therapeutics, in the Faculty of Medicine, became vacant. According to the practice on these occasions, the vacant chair was thrown open to be filled by a *concours*. The *concours* for a full professorship, it must be borne in mind, is open only to *agregés* (adjuncts) of the Faculty of Medicine, and of the Hospitals—men necessarily already distinguished for scientific attainments. Upon these occasions, one of the trials of the competitors consists in the composition of a lecture, upon a given subject, after twenty-four hours' preparation,—that is to say, the subject of the lecture is announced at 4 o'clock in the evening, the lecture to be completed by the same hour the succeeding day. In my opinion, of all the trials incident to the *concours*, this is the best test of ability, as it, in fact, comprises a discharge of the precise duties of a professorship. A professor may fairly be supposed capable of working out a lecture on his speciality, after a day's preparation, particularly in a city like Paris, where he has at hand to consult both the men and books to whom he may wish to have access.

To Dr. Trousseau, one of the competitors for the vacant chair of M. Alibert, was assigned a subject for a lecture, alike difficult and interesting—namely, the system of counter-stimulation, and the principal therapeutic agents employed in it. I shall, I think, be presenting to your readers acceptable matter, by detailing, as faithfully as possible, the substance of his lecture.

The doctrine of counter-stimulation is the offspring of the doctrine of Brown, overthrown by Broussais and by Tomasini; for Rasori has merely reproduced the ideas of Brown, with a change of the terms employed by the latter. The substance of this doctrine is comprised in the four following propositions:—1st, there exist both sthenic and asthenic diatheses; 2d, the physician should not allow his attention to be fixed by the idea of local lesions; 3d, all agents, which exert an influence upon the human economy, are either stimulant, or counter-stimulant; 4th, the nature of the diathesis is decided upon, not from the symptoms present, but from the result of the treatment. These are the fundamental axioms of Tomasini. He, moreover, considers in the patient the organ which is the seat of the stimulus, which he denominates the *support of the stimulus;* the stimulus, or agent itself; and, finally, the aptitude of the organ to be influenced by the stimulus, which aptitude takes the name of *incitability*. The regular influence of a normal agent is styled *physiological power;* the abnormal action of a morbific agent, *pathological power*. When, adds Tomasini, an external stimulus puts into action the incitability of an organ, the result is a phlogosis, and this phlogosis is made general by diffusion. It becomes *universally local*, to use the author's own expression: thus, the virus of variola, applied to the arm, produces at first a local phlogosis; it is thence propagated to all the molecules of the body, the disease is repeated as many times as there are molecules, and becomes general, or, as the advocates of the counter-stimulant doctrine express it, universally local. They, however, admit the existence of sympathetic, or dependent affections; as, for example, when an individual has a calculus engaged in the urethra, and is seized with vomiting. Like all the pure solidists, the Italians deny the existence of special stimulants, as the virus of syphilis, variola, rabies, &c. Just, however, as the wounds inflicted by different insects upon trees produce galls differing in appearance, but always the same for each variety of insects, there must, in like manner, exist special stimulants for the animal economy. It will be seen that the doctrine above described differs essentially from that of Broussais. The latter always admits a local lesion, or affection, and the general symptoms are but the result of the sympathy of other organs with that which is suffering. The therapeutic consequences to which these two doctrines lead, are likewise essentially different. Broussais treats the local affection, Tomasini combats the general symptoms.

Let us now examine how the Italian school understands the action of counter-stimulant remedies. They act, say they, by modifying the incitability. It matters little what be the nature of the morbific agent, we have only to do with the reaction which we are endeavouring to operate in the economy. The number of counter-stimulant agents is considerable. It comprises, in the first place, all the antiphlogistics, the antimonials, narcotics, emetics, purgatives, tonics, sedatives, bark, the mercurials, in a word, all the powerful remedies of the materia medica. All these agents, according to Tomasini, act in the same diseases by modifying the general incitability; he does not recognise in them any special action. Some, however, of his disciples admit that bleeding and tartar emetic are more particularly appropriate in pneumonia; the solanum, aconite, and colchicum, in neuralgia.

It remains for us to examine how the counter-stimulant hypothesis accounts for the influence of agents so diversified, and so often opposite in their action. Let us first examine the influence of bleeding.

The blood is a nutritious, or supporting element, when it stimulates an organ normally, and communicates to it a certain degree of normal and regular excitability. If the nutritious power is deficient, the excitability is diminished; if it be in excess, the latter is increased. Thus, in a normal state, the eye, when exposed to the action of the light, is stimulated normally; bleed a man freely, and there will be disturbance of vision, because the incitability is dimished. But, it is said, since the application of cold determines a temporary anemia in an organ by the abstraction of its incitability, it ought to be employed in the more serious inflammations. In fact, Compagnaro did not hesitate to follow out this practical consequence of the doctrine, and recommended the cold bath in pneumonia. Broussais regards bleeding in quite a different point of view. By abstracting a certain quantity of blood, he diminishes the stimulus, and *modifies* the incitability. The counter-stimulists deny the possibility of modifying or altering this fluid, and insist that the disease is seated exclusively in the nervous fibres; another consequence of their doctrine is the necessity of bleeding as long as the fever, that is to say, the incitability, persists. Yet, what prudent physician will not stop, when the frequency and fulness of the pulse have begun to yield! What observing physician is unaware of the ability of nature to finish the cure, and of the greater promptness of convalescence, when her efforts are not crippled!

The counter-stimulant doctrines have been introduced into France by the use of the antimonials; and most physicians suppose that these preparations are favourite remedies with the Italian sectaries. Tomasini, however, declares that their action is very inferior to that of bleeding and mercurials. He recommends them in pneumonia, but in angina he prefers copious bleedings. Rasori, the great advocate of tartar emetic, says that it destroys the incitability, because the action of the pulse is enfeebled, the patient becomes pale, and sometimes falls into syncope. Wepfer relates the case of a woman, who, after having taken a considerable dose of tartar emetic to procure abortion, fell into a state of syncope, and lost a toe and finger from gangrene. Another woman having swallowed some colocynth for a similar purpose, suffered from gangrene of the foot. Barbier was witness of another such fact, in which the extremities and an ear fell off, after having lost their vitality. All these cases the counter-stimulists view as so many facts, incontestibly proving the anti-incitable action of tartar emetic. The French physicians do not deny the good effects of tartar emetic in pneumonia, but they explain them upon the principle of revulsion; looking upon them as analogous to the effect of cups to the nucha in an ophthalmia. The question has been asked the Bolognese physicians in what manner they determine the dose necessary entirely to extinguish the morbid incitability of an organ. Rasori replies by coining the word *tolerance.* So long, says he, as tartar emetic does not produce vomiting, the incitability continues to exist; following out this principle, half an ounce of tartar emetic has been administered in a single day. The falseness of this theory is proved by the fact that Dr. Trousseau has seen tartar emetic perfectly tolerated by persons affected with chronic rheumatism and ophthalmia without fever, and on the other hand borne very ill by others affected with serious inflammations. It must, however, be admitted that tolerance is usually met with in the latter cases. When it ceases, the Italians say that an *antimonial saturation* has taken place—a saturation analogous to that which is recognised in the case of mercurials, when a patient shows the first symptoms of salivation. The antimonial saturation shows itself by the eruption of pustules upon the tongue, lips, and palate. But, objects Dr. Trousseau, this eruption may be produced at pleasure,—upon the skin with the ointment; in the mouth, by administering doses of the tartar emetic, at short intervals, without washing the mouth. When tartar emetic is administered at long intervals, without resorting to the precautions hereafter marked out, the phenomenon in question never takes place, no matter how far the doses of the medicine may be pushed.

Mercurials play a conspicuous part in the novel system of Italian medicine. The French readily admit their efficacy in peritonitis. The English and Americans give calomel in almost all inflammatory affections, and it is difficult to suppose that a practice so universal can be decidedly bad. Dr. Trousseau has seen a third of the cases of rheumatism treated by him yield to calomel pushed to salivation. In fact, who can doubt the powerful action of mercury! A grain of corrosive sublimate in a gallon of water, prevents the development of all organized matter, vegetable or animal. Eggs placed in contact with mercurial vapour, cease to be hatched, notwithstanding the continued incubation of the hen. Tomasini admits the efficacy of mercury in destroying incitability. It is thus that he explains its anti-syphilitic power, and yet how many cases of secondary symptoms which yield to mercury, would never have been relieved by bleeding. Dr. Trousseau is, however, convinced that mercury exercises an action upon the coagulability of the blood. In support of his opinion, he cites the following facts. A patient was affected with acute rheumatism; a bleeding was ordered, the blood of which was strongly sized. During the day, frictions were made with mercurial ointment, and in the evening the blood had ceased to be sized. Again,—the keeper of the bridge of Montereau came to Paris, to take advice for a severe pain of the thigh. Dr. Sanson, taking it to be a sciatica, prescribed sixty leeches, afterwards a blister, and moxas. At the end of five days, there was no amelioration of the symptoms. Dr. Trousseau, who was then consulted, observed a tumefaction on the tibia. He recommended mercurial frictions, after three days' use of which salivation took place, when all the leech-bites, without a single exception, reopened and bled so freely, that it became necessary to resort to the most powerful anti-hæmorrhagic remedies.

